# Supplementary material for: PPM1B utilizes a trinuclear metal architecture for phosphatase activity
Source: bioRxiv. 2026 Apr 27:2026.04.23.720145. Preprint. [Version 1] doi: 10.64898/2026.04.23.720145 (PMC13142519; doi:10.64898/2026.04.23.720145)
Supplement: Supplement 1 [file NIHPP2026.04.23.720145v1-supplement-1.pdf]

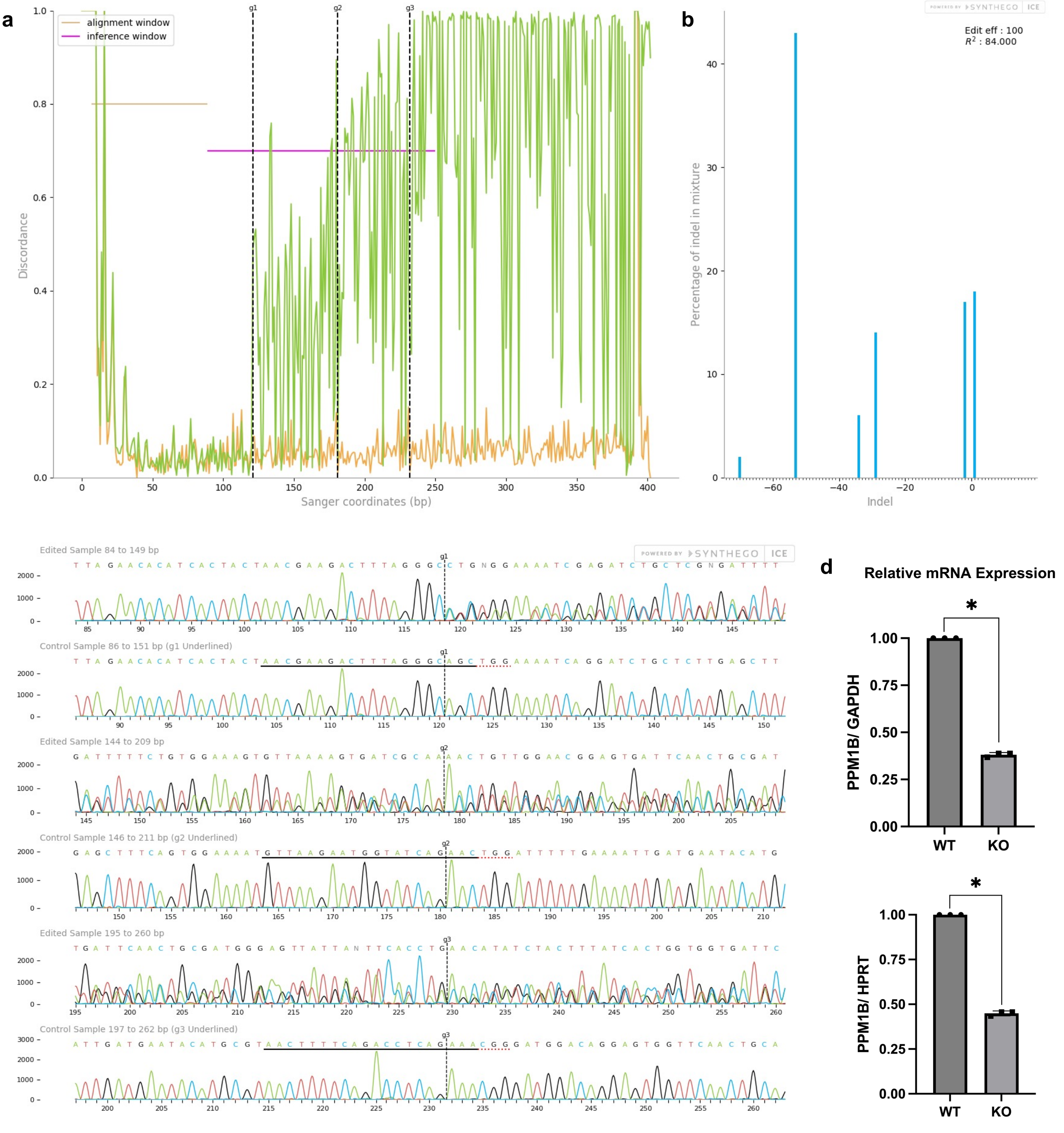

Supplementary Fig. 1 | Validation of PPM1B knockout in A549 cells. a, Discordance plot comparing Sanger sequences of WT (orange) and CRISPR KO (green) cells. Dotted black lines indicate guide RNA cut sites (g1–g3). **b**, Indel plot showing frequency and size distribution of insertions/deletions in KO cells. **c**, Representative Sanger traces of WT and KO cells at guide RNA target sites; black underline = guide sequence; dotted red line = PAM; dotted vertical line = cut site. **d**, Quantitative PCR showing PPM1B mRNA levels normalized to GAPDH and HPRT.

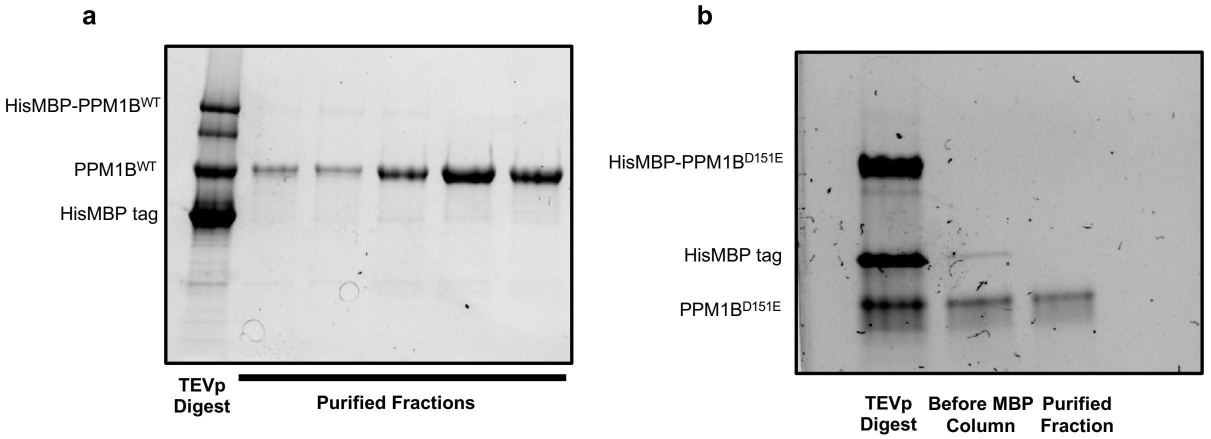

Supplementary Fig. 2 | Purification of recombinant human PPM1B proteins. a, b, Gels showing TEV protease (TEVp) digestion and final purified fractions of (**a**) wild type PPM1B (PPM1B^{WT}) and (**b**) PPM1B with a D151E mutation (PPM1B^{D151E}) after size-exclusion chromatography.

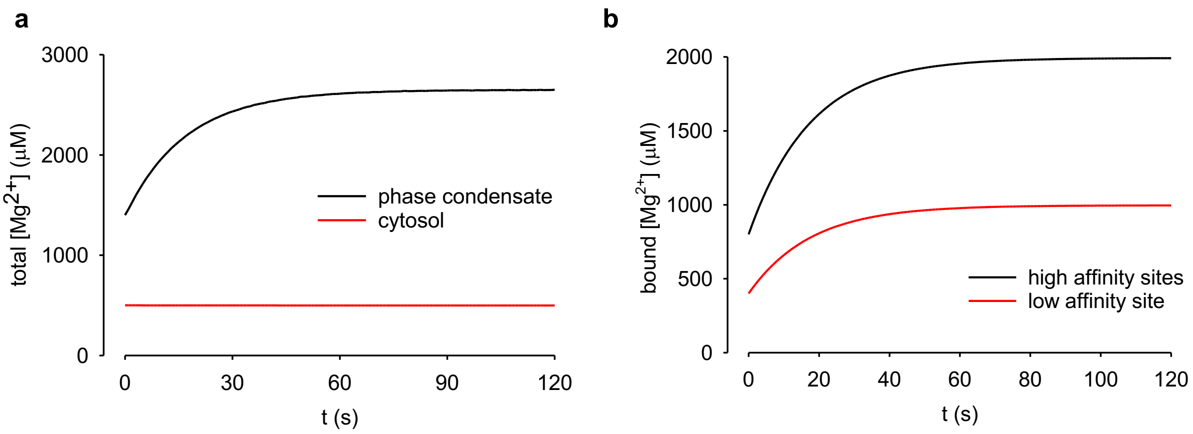

c

Mg^{2+} Binding	Condensate (C1)	C1 % occupied	Cytosol (C2)
Total Mg^{2+}	2.8 mM	--	0.5 mM
Bound to high-PPM1B affinity site 1 (M1)	990 µM	100%	--
Bound to high-PPM1B affinity site 2 (M2)	990 µM	100%	--
Bound to low-affinity PPM1B site 3 (M3)	440 µM	50%	--
Unbound Mg^{2+}	380 µM	--	400 µM
Bound to competing macromolecules	--	--	100 µM

Supplementary Fig. 3 | Simulation of Mg^{2+} distribution in cytosol and liquid-liquid phase condensates. a, Modeled distributions of total Mg^{2+} in the cytosol (red) and phase condensates (black). Kinetics are dominated by diffusion constraints at the condensate-cytosol interface. **b**, Mg^{2+} binding kinetics to low- and high-affinity PPM1B sites within the phase condensate. High-affinity sites bind faster than cytosolic flux. **c**, Occupancy of Mg^{2+} at high- (M1 and M2) and low-affinity (M3) sites in a 100 nm sized condensate with 1 mM PPM1B. High-affinity sites are fully occupied, low-affinity sites reach ~50% occupancy under modeled conditions.

Supplementary Table 1 | Parameter definitions, values, and initial conditions used to simulate Mg^{2+} sequestration into the PPM1B phase condensate

variable	description	value	initial condition	steady state
V1	volume of compartment 1, the phase condensate	0.002 pL	–	–
V2	volume of compartment 2, the bulk cytosol	2.0 pL	–	–
n1	total [Mg^{2+}] in the phase condensate	–	1.40 * 10^{-3} M	2.65 10^{-3} * M
n2	total [Mg^{2+}] in the cytosol	–	0.50 * 10^{-3} M	0.50 * 10^{-3} M
n3	[PPM1B] without Mg^{2+} bound to high affinity binding site 1	–	0.70 * 10^{-3} M	0.63 * 10^{-3} M
n4	[PPM1B] with Mg^{2+} bound to high affinity binding site 1	–	0.30 * 10^{-3} M	0.37 * 10^{-3} M
n5	[PPM1B] without Mg^{2+} bound to high affinity binding site 2	–	0.60 * 10^{-3} M	0.00 * 10^{-3} M
n6	[PPM1B] with Mg^{2+} bound to high affinity binding site 2	–	0.40 * 10^{-3} M	1.00 * 10^{-3} M
n7	[PPM1B] without Mg^{2+} bound to low affinity binding site	–	0.60 * 10^{-3} M	0.00 * 10^{-3} M
n8	[PPM1B] with Mg^{2+} bound to low affinity binding	–	0.40 * 10^{-3} M	1.00 * 10^{-3} M
n9	[high affinity cytosolic buffer] without Mg^{2+} bound	–	0.10 * 10^{-3} M	0.00 * 10^{-3} M
n10	[high affinity cytosolic buffer] with Mg^{2+} bound	–	0.90 * 10^{-3} M	0.10 * 10^{-3} M
n11	[low affinity cytosolic buffer] without Mg^{2+} bound	–	1.90 * 10^{-3} M	1.89 * 10^{-3} M
n12	[low affinity cytosolic buffer] with Mg^{2+} bound	–	0.10 * 10^{-3} M	0.11 * 10^{-3} M
k1	Mg^{2+} flux coefficient from the phase condensate into the cytosol	0.01*10^{-6} L/s	–	–
k2	Mg^{2+} flux coefficient from the cytosol into the phase condensate	0.01*10^{-6} L/s	–	–
k_{on1}	rate constant for Mg^{2+} binding to low affinity PPM1B binding sites	200 $M^{-1}s^{-1}$	–	–
k_{off1}	off rate for the low affinity PPM1B binding site	0.1 s^{-1}	–	–
k_{on2}	rate constant for Mg^{2+} binding to high affinity PPM1B binding sites	200 $M^{-1}s^{-1}$	–	–
k_{off2}	off rate for high affinity PPM1B binding sites	2*10^{-4}	–	–
k_{on3}	rate constant for binding to high affinity cytosolic Mg^{2+} binding sites	200 $M^{-1}s^{-1}$	–	–
k_{off3}	off rate for the high affinity cytosolic Mg^{2+} binding sites	2*10^{-4}	–	–
k_{on4}	rate constant for Mg^{2+} binding to low affinity cytosolic binding sites	200	–	–
k_{off4}	off rate for Mg^{2+} binding to low affinity cytosolic sites	1 s^{-1}	–	–
